# Strength of Intentional Effort Enhances the Sense of Agency

**DOI:** 10.3389/fpsyg.2016.01165

**Published:** 2016-08-03

**Authors:** Rin Minohara, Wen Wen, Shunsuke Hamasaki, Takaki Maeda, Motoichiro Kato, Hiroshi Yamakawa, Atsushi Yamashita, Hajime Asama

**Affiliations:** ^1^Department of Precision Engineering, The University of TokyoTokyo, Japan; ^2^Department of Neuropsychiatry, Keio University School of MedicineTokyo, Japan

**Keywords:** sense of agency, intentional effort, self-attribution task, cue integration, comparator model

## Abstract

Sense of agency (SoA) refers to the feeling of controlling one’s own actions, and the experience of controlling external events with one’s actions. The present study examined the effect of strength of intentional effort on SoA. We manipulated the strength of intentional effort using three types of buttons that differed in the amount of force required to depress them. We used a self-attribution task as an explicit measure of SoA. The results indicate that strength of intentional effort enhanced self-attribution when action-effect congruency was unreliable. We concluded that intentional effort importantly affects the integration of multiple cues affecting explicit judgments of agency when the causal relationship action and effect was unreliable.

## Introduction

When we intend and take actions, a subjective feeling that we have caused those actions and their effects usually arises. Sense of agency (SoA) refers to this subjective feeling of controlling one’s own actions and causing external events (Gallagher, 2000). SoA is a crucial element of self-consciousness; many fields (e.g., psychology, cognitive neuroscience, and psychiatry) have accordingly examined it. SoA’s underlying mechanisms remain controversial, however. For example, the comparator model suggests that internal predictions of sensory feedback are generated from an efference copy of a motor signal, and are compared with actual sensory feedback: SoA arises if the copy and the feedback match, and is inhibited if they do not (Frith et al., 2000). Recent studies, however, have suggested that SoA is determined not only by internal forwarding mechanisms, but also by an integrative process involving both internal and external cues. Internal cues are mental states (e.g., motor signals, priming, beliefs, knowledge, effort, and reward expectation); in contrast, external cues are perceived information (e.g., effect, contextual information, and actual reward). Cue integration theory thus proposes that SoA is generated from the integration of multiple internal and external cues, whose weightings are determined by their availability and reliability (Moore and Fletcher, 2012; Synofzik et al., 2013).

In terms of the internal cues of cue integration theory, recent empirical studies have shown that subliminal priming prior to an action influenced SoA (Chambon and Haggard, 2012; Sidarus et al., 2013; Damen et al., 2014). However, among the internal cues influencing experience of SoA, effort has received comparatively little attention. Thus, in the present study, we examined the effect of strength of intentional effort on SoA.

Individuals are considered to expend effort when they cause actions using their bodies (Preston and Wegner, 2009); the sensation of effort is considered to accompany the performance and control of actions (Lafargue and Franck, 2009). One previous study investigated the effect of effort on intentional binding, which is the implicit measure of SoA (Demanet et al., 2013). In their experiment, participants performed the intentional binding task using a PC mouse with their right hand while pulling a stretch band of varying resistance levels. However, the manipulation of effort in this study was not related to actions that caused external events. Hence, no study has yet examined the effect of effort strength that is directly linked to external events on the processes underlying SoA (we refer to this as “intentional effort”). We consider that using actions to cause external events is an important element of understanding the roles of effort in SoA experiments. Indeed, according to the “ideomotor theory,” what we intend to cause with our actions influences how we move our body (Pfister et al., 2010, 2014a,b). Thus, in the SoA experimental paradigm, intentional effort—which is directly linked to action effects—must be introduced.

In previous research, two types of experimental tasks have been used to examine SoA: subjective SoA judgment tasks and intentional binding effect tasks. In tasks examining subjective judgments of SoA, participants are instructed to make an explicit judgment of self- or other-attribution. For example, participants may report on whether they judged they had caused a certain effect (Aarts et al., 2005; Kühn et al., 2011; Maeda et al., 2012, 2013). Intentions, thoughts, and social and contextual cues likely importantly affect such explicit judgments of agency (Synofzik et al., 2008). In contrast, the intentional binding effect is the temporal compression of the perceived interval between voluntary actions and their sensory consequences (Haggard et al., 2002; Haggard, 2005). In the original paradigm of the intentional binding effect, participants were instructed to judge the onsets of either their voluntary actions, or tones presented 250 ms after the actions. Compared to a baseline condition in which participants’ actions did not cause tones or tones were presented without actions, participants’ judged time of their actions’ onset shifted toward the tones, and participants’ onset judgments of tones shifted toward the actions. The intentional binding effect is not equal to SoA (Ebert and Wegner, 2010), but is considered to reflect implicit aspects of SoA (Moore et al., 2012).

In this study, we used an agency attribution task to determine how delayed effect and intentional effort—as external and internal factors, respectively—as well as their interaction influenced SoA emergence. We hypothesized that stronger intentional effort (i.e., an internal cue) would promote SoA under conditions wherein visual feedback (external cue) was unreliable. This hypothesis was based on Moore and Fletcher’s (2012) cue integration theory, which suggests that SoA emergence is compensated via internal cues when external cues are unreliable due to the temporal incongruence of action and feedback. We used a simple action–feedback task modified from previous studies, in which participants pushed a button to cause changes in an object (Maeda et al., 2012, 2013), and manipulated the delay before the change to create unreliable conditions. Further, we manipulated effort strength using three types of button requiring different pushing force. Pushing a button requiring more force (a “harder” button), was considered to involve stronger effort than pushing a button requiring less force (a “softer” button); participants were informed of the types of button before trials.

## Materials and Methods

### Participants

Twenty-five students from the University of Tokyo participated in the study (six female, mean age 22.7, *SD* = 1.71). The present study was approved by the ethics committee at the Graduate School of Engineering of the University of Tokyo; all participants gave written informed consent before participating.

### Intentional Effort Manipulation

The strength of intentional effort was manipulated with three different buttons (OMRON VX-5-1A2, V-10-1A4, V-15-1A6) requiring different amounts of pushing force. The three buttons had the same appearance and stroke, and differed only in the force required to depress them. The required amounts of force were 0.10, 0.65, and 2.70 N for the soft, medium, and hard buttons, respectively. The soft, medium, and hard buttons were used in the weak, medium, and strong intentional effort conditions, respectively.

### Task

**FIGURE 1:**
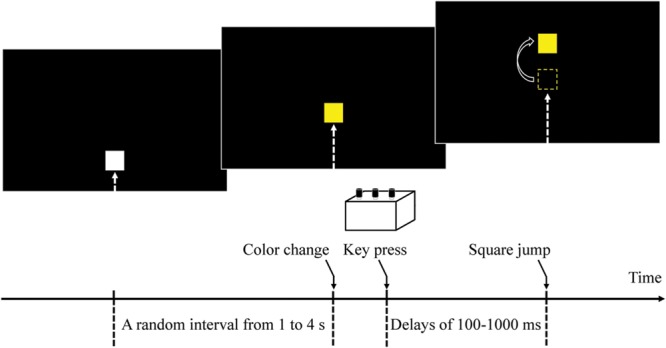
**A timeline of a trial.** Each trial started with a 180 mm × 245 mm black screen, and with a 5-mm white square which appeared from the bottom of the screen and moved upward at a speed of 20 mm/s. Participants were instructed to push a specified button as quickly as possible with their dominant index finger when they saw the color of the square change to yellow. After pushing the button, the square jumped 25 mm upward at a random delay (100, 200, 300, 400, 500, 700, or 1000 ms).

In the self–other attribution task (**Figure 1**), each trial began with a 180 mm × 245 mm black screen, and with a 5 mm white square, which appeared from the bottom of the screen and moved upward at a speed of 20 mm/s. Participants were instructed to push one of the three buttons (buttons were consistent in each block and differed between blocks) as quickly as possible with their dominant index finger when they saw the square’s color change to yellow. After participants pushed the button, the square jumped 25 mm upward at a random delay (100, 200, 300, 400, 500, 700, or 1000 ms). Participants were informed that the computer would sometimes interrupt their commands, and cause the box to jump after an arbitrary delay. After the square had jumped, participants orally reported whether they felt they had caused the square to jump upward as intended by giving “yes” or “no” responses.

### Procedure

Participants were tested individually in a quiet room. After receiving an explanation of the experimental tasks, participants pushed each button 10 times to acquaint themselves with the buttons’ hardness. The task used three blocks, and each block used one of the three buttons. Before each block, participants performed three practice trials with the appropriate button to acquaint themselves with that button’s hardness. In each block, participants performed 70 trials, comprising 10 trials for each delay condition. Trial order was randomized in each block. Block order (i.e., order of buttons) was counterbalanced between participants. The experiment took 45 min on average.

## Results

**FIGURE 2:**
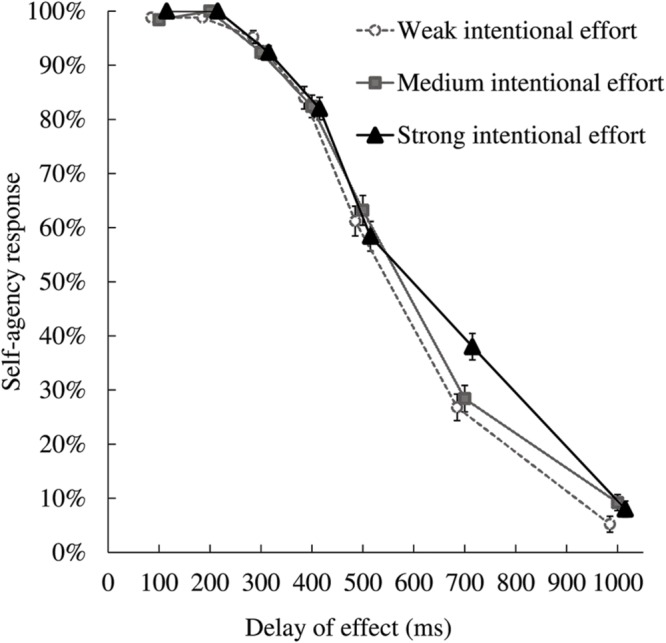
**Mean proportions of self-responses in the self–other attribution task.** Error bars represent within-subject standard errors for each delay condition, as computed according to Loftus and Masson’s (1994) method. The self-agency response rate decreased with increasing delay, and was greater in the strong intentional effort condition than in the weak and medium intentional effort conditions when the delay of effect was 700 ms.

The mean proportions of yes-responses (i.e., self-agency responses) for each condition are shown in **Figure 2**. Self-attribution decreased with increasing delay. We focused on the delay conditions in which visual feedback was unreliable. For statistical analysis, we first checked the normality of the results, and then conducted parametric tests on those results that were well-modeled by the normal distribution, and non-parametric tests for those that were not.

We used the Kolmogorov–Smirnov test for each delay and intentional effort condition to check the normality of the data. The results in the 500 and 700 ms delay conditions in all the intentional effort conditions were well-modeled by the normal distribution (for the weak condition, *d* = 0.127 and 0.200; for the medium condition, *d* = 0.177 and 0.199; for the strong condition, *d* = 0.195 and 0.202, respectively, *n.s.*). However, the results from other delay conditions (100, 200, 300, 400, and 1000 ms) were not normally distributed.

Regarding the 500 and 700 ms delay conditions, we conducted a 3 × 2 (intentional effort × delay) repeated-measures ANOVA on participants’ responses; regarding other delay conditions, we used Friedman’s test for further analysis. Regarding the ANOVA, the main effect of delay was significant, but the main effect of intentional effort was not [*F*(1,24) = 55.05, *p* < 0.01, η_p_^2^ = 0.696; *F*(2,48) = 0.91, *n.s.*, η_p_^2^ = 0.036, respectively]. The interaction between intentional effort and delay was significant [*F*(2,48) = 9.00, *p* < 0.01, η_p_^2^ = 0.273]. *Post hoc* testing (Bonferroni corrected) of the interaction indicated that self-attribution was significantly elevated in the strong intentional effort condition compared to the weak and medium intentional effort conditions when the delay was 700 ms (*p* < 0.01; *p* < 0.05, respectively), but did not differ between intentional effort conditions in the 500 ms delay condition. Regarding Friedman’s test, in all delay conditions whose results were not normally distributed, participants’ responses did not differ significantly between the intentional effort conditions (100 ms: χ^2^(2) = 3.714, *n.s*.; 200 ms: χ^2^(2) = 6.000, *n.s.*; 300 ms: χ^2^(2) = 2.480, *n.s.*; 400 ms: χ^2^(2) = 0.237, *n.s.*; 1000 ms: χ^2^(2) = 3.561, all *n.s.*).

## Discussion

In the present study, we investigated the effect of intentional effort on self-agency judgment. The self-attribution task was an explicit measure of SoA. We manipulated the strength of intentional effort using three types of button requiring different amounts of pushing force. Intentional effort was considered stronger when participants pushed the hard button compared to the medium and soft buttons. We hypothesized that stronger intentional effort would promote SoA in the condition in which visual feedback was unreliable. With a simple action-feedback task, we found a significant effect of intentional effort when feedback was delayed by 700 ms and the ratio of self-agency dropped to 30–40%. These results indicate that self-attribution was significantly increased by increased effort strength only when the reliability of action feedback was very low.

Previous research has reported that judgments of self-attribution are inhibited by long temporal delays of actions’ consequences (Sato and Yasuda, 2005; Ebert and Wegner, 2010; Kühn et al., 2011; Maeda et al., 2012, 2013; Farrer et al., 2013; Hon et al., 2013; Kawabe, 2013; Wen et al., 2015). In other words, when an action’s consequence follows a considerable delay, people tend to attribute the consequence to others, rather than themselves. Our results replicated this finding. The proportion of self-agency responses decreased to around 10% across all types of button when the delay was 1000 ms; however, this tendency was affected by strength of intentional effort in the 700 ms delay condition. Specifically, when participants pushed the hard button, their self-agency responses decreased less compared to in conditions where they pushed softer buttons with about 700 ms delay. The 700 ms delay condition was the only condition in which self-agency responses were affected by intentional effort. It is possible that when the visual feedback delay became sufficiently long that self-agency responses decreased to around 30–40%, visual feedback became most unreliable and responses were most strongly affected by the strength of intentional effort. In this condition of uncertain causality, judgment of agency may be more easily affected by other factors, such as intentional effort, thoughts, and social and contextual cues. In the present study, participants who pushed the hard button likely had stronger intentional effort than participants who pushed the medium or soft buttons; this stronger intentional effort may have contributed to participants’ self-attribution judgment, explaining the more frequent self-agency responses. This would suggest that when the causal relationship between actions and effects was less unreliable (i.e., when the delay was shorter or longer than 700 ms), intentional effort did not affect judgment of agency. Although this difference may seem subtle, we believe that it may be critical in the SoA experience, given that it emerges at a millisecond-level time window. However, because the limited number of possible trials, we did not test delays between 500 and 700 ms. Therefore, it was not possible to determine the exact point of reliability at which intentional effort’s effect began to appear. Future research should identify this point.

Our results were consistent with the cue integration theory of SoA (Moore and Fletcher, 2012), which was proposed to account for SoA’s emergence. That article suggested that SoA depends on both internal and external cues, and that the integration of these cues are affected by each cue’s reliability. When a predicted effect occurs immediately after an action, or in contrast, obviously delayed from an action, motor signals, and the following effects are considered to have more weight than other cues in the judgment of agency (Moore and Fletcher, 2012); however, when the causal relationship between actions and effects becomes unreliable, other cues besides motor signals and predictions may have a greater effect. In the present study, we manipulated an internal cue—strength of intentional effort—and found that it significantly affected judgment of self-attribution only when the causal relationship between action and effect became as unreliable as the condition in which self-agency responses decreased to around 30–40%.

Although the sensation of effort has been considered to accompany action and action control, no research had examined the effect of intentional effort on SoA. Prior study demonstrated the effect of effort not related to the event-causing action (Demanet et al., 2013). Our results provide the first evidence that strength of intentional effort affects the explicit aspects of SoA. In the present study, however, tactile feedback in the hard button condition was stronger than in the medium and soft conditions, in addition to stronger intentional effort. This may also affect judgments of agency, and should be examined in future research. Moreover, although we manipulated an internal cue in this study, it is possible that the intentional effort had an effect on other internal cues, such as motor signals and prediction of feedback; as such, this point should be examined in future studies.

Our results are also important regarding engineering. The feeling of control is indispensable to people manipulating a human–machine interface; this feeling is precisely SoA. A good feeling of SoA is particularly important in operating remote–control interfaces; however, operating delay is often inevitable in this setting. Our results indicate that operating effort may assist operators who experience such delays.

## Conclusion

We demonstrated that strong intentional effort promotes judgments of self-attribution when the causal relationship between actions and effects is uncertain. Intentional effort strength’s effect is likely greater when action-effect congruency is unreliable. This study may contribute to the understanding of the underlying mechanisms of SoA. Further, we recommend that future studies examine intentional effort’s effect on SoA in psychopathological conditions such as schizophrenia.

## Author Contributions

RM, WW, SH, TM, MK, HY, AY, and HA conceived and designed the experiments. RM performed the experiments. RM and WW analyzed the data. RM, WW, SH, TM, MK, HY, AY, and HA discussed the results and wrote the manuscript.

## Conflict of Interest Statement

The authors declare that the research was conducted in the absence of any commercial or financial relationships that could be construed as a potential conflict of interest.
